# Candesartan, Metoprolol and Rosuvastatin Associated to Lower 30-Days Mortality in Adult COVID-19 Patients – A Register Study in Finland before COVID-19 Vaccines

**DOI:** 10.1177/21501319261453019

**Published:** 2026-05-21

**Authors:** Anna Hyvärinen, Mika Helminen, Markku Broas, Hannu Syrjälä

**Affiliations:** 1 Department of Surgery, Tampere University Hospital, Wellbeing Services County of Pirkanmaa, Tampere, Finland; 2 Faculty of Medicine and Health Technology, Tampere University, Tampere, Finland; 3 Department of Surgery, Länsi-Pohja Central Hospital, Wellbeing Services County of Lapland, Kemi, Finland; 4 Department of Pediatric Surgery, 60664 Oulu University Hospital, Wellbeing Services County of Northern Ostrobothnia, Oulu, Finland; 5 Faculty of Medicine, University of Oulu, Oulu, Finland; 6 Faculty of Social Sciences, Health Sciences, Tampere University, Tampere, Finland; 7 Tays Research Services, Tampere University Hospital, Wellbeing Services County of Pirkanmaa, Tampere, Finland; 8 Department of Internal Medicine, Länsi-Pohja Central Hospital, Wellbeing Services County of Lapland, Kemi, Finland; 9 Department of Internal Medicine, Infection Control Unit, Lapland Central Hospital, Wellbeing Services County of Lapland, Rovaniemi, Finland; 10 Division of Intensive Care Medicine, Research Group of Surgery, Anesthesiology and Intensive Care Medicine, 60664 Oulu University Hospital, Wellbeing Services County of Northern Ostrobothnia, Oulu, Finland

**Keywords:** COVID-19, prescription drugs, mortality, candesartan, metoprolol, rosuvastatin

## Abstract

Elderly people with chronic diseases are at risk for having severe COVID-19 disease. We were interested whether the drugs prescribed for the chronic diseases are associated with lower mortality in COVID-19 patients.

We used Finnish national registry data for analysis of potential associations of 73 prescription drugs to mortality among 6082 adult COVID-19 patients between 1.1.2020 and 30.6.2020, before COVID-19 vaccinations were available. Adjusted odds ratios (aORs) with 95 percent confidence intervals (95% CIs) for different medications are presented.

The use of three cardiovascular drugs was associated with lower 30 days all-cause mortality rate: candesartan (aOR 0.34, 95% CI 0.16-0.72, p=0.005), metoprolol (aOR 0.37, 95% CI 0.16-0.88, p=0.024) and rosuvastatin (aOR 0.44, 95% CI 0.21-0.94, p=0.034).

In conclusion, COVID-19 patients who used cardiovascular drugs candesartan, metoprolol or rosuvastatin have lower 30 days all-cause mortality than other COVID-19 patients.

## Introduction

The SARS-CoV-2 is a single-stranded RNA virus, which have a strong tendency to develop new mutations in each replication cycle.^1^ Due to the tendency of the SARS-CoV-2 to develop new variants as well as the fact that the virus can infect several animal species in addition to humans,^2^ the eradication of the SARS-CoV-2 virus seems unlikely. Intensive research and development of vaccines as well as drugs to treat severe COVID-19 has occurred.^3-5^ In addition, repurposing of the existing drugs to treat, prevent or mitigate COVID-19 disease has been in the focus of the research.^3,4^ Important improvements for the treatment of severe COVID-19 infection have been made. In the USA, the Centers for Disease Control and Prevention recommends considering COVID-19 treatment in patients with mild or moderate COVID-19 who have one or more risk factors for severe COVID-19 to reduce progression to hospitalization and/or death. There are several possibilities for the treatment of COVID-19 infection, which vary depending on the severity of the disease.^5^ These include, for example, ritonavir-boosted nirmatrelvir, intravenous remdesivir, or in selected cases dexamethasone as well as pre- and post-exposure prophylaxis.^6^

Old age, male sex as well as several chronic diseases are associated to severe COVID-19 disease and increased mortality. Diseases associated to higher COVID-19 mortality include cardiovascular diseases, hypertension, diabetes, COPD, malignancy, cerebrovascular diseases and chronic kidney diseases.^7^ Also, immunocompromised patients, those with chronic liver disease, psychotic disease, epilepsy or neurologic disease affecting breathing, severe chronic lung disease, sleep apnoea and severe obesity are associated with severe COVID-19 disease.^8-11^ Whilst the immunity of population against COVID-19 has generally improved due to vaccination programs and acquired COVID-19 infections, especially elderly people with chronic diseases are still at risk to have severe COVID-19 disease. These people are often using multiple drugs, and we do not know much on the effects of these drugs on the course of COVID-19 infection. According to previous studies, some of the common prescription drugs could have potential to lower the risk of severe COVID-19 disease and related mortality. Particularly, angiotensin-converting enzyme inhibitors and angiotensin receptor blockers have been studied for their potential to reduce COVID-19 mortality. A large study from Spain analysed these drugs in a real-world setting and found that candesartan and enalapril could reduce severity of COVID-19 disease as well as related mortality.^12^

The present study was designed to evaluate the potential associations of different prescription drugs to lower COVID-19 mortality in Finnish population before introduction of COVID-19 vaccinations.

## Methods

This register study is based on data extracted from the Finnish National Infectious Diseases Register upheld by the Finnish Institute of Health and Welfare and the Register of Reimbursed Drug Purchases upheld by the Finnish Social Insurance Institution (Kela). The COVID-19 patients were identified from the Finnish National Infectious Diseases Register, including all PCR-test confirmed COVID-19 cases within the study time frame, 1.1.2020-30.6.2020. The hospitals and other health care units had a statutory reporting obligation. After identifying the patients, the data on their prescription drug purchases was collected from the Register of Reimbursed Drug Purchases and information of the day of death was collected by Statistics Finland. The data on all prescription drug redemptions in Finland are automatically transferred to the national Register of Reimbursed Drug Purchases. The data collection from registers was done by the employees of the register keepers as only they have the access to these registers. The data from different registers was combined for this study by Findata. During the study, the data was stored, and the analyses were carried out in the Findata data user secure environment via remote access connections. As this is a register study and we did not contact the patients, the requirement of patient´s written consent was waived.

The national register data was available for 6707 PCR-confirmed COVID-19 cases in Finland, of which 168 were excluded due to prohibition of data disclosure, missing information on municipality of residence or living abroad on index date (1.1.2020), incorrect social security number or age below 18 years on index date. Subsequently, the study cohort included 6082 PCR-confirmed COVID-19 cases between 1.1.2020 and 30.6.2020. Of these, 4582 cases were found from the Register of Reimbursed Drug Purchases and had drug purchases that met the criteria for drug exposure. These patients were included in the analyses. We studied the mortality of the COVID-19 positive users of each prescription drug to investigate if there was association between the drug usage and lower mortality among the COVID-19 patients. This was compared to mortality of the COVID-19 positive non-users of the analysed drug. The mortality was defined as 30 days all-cause mortality (all deaths within 30 days from positive COVID-19 test) between 1.1.2020 and 30.7.2020. The drug exposure was defined as purchase of the drug after 31.12.2019 and before or within 30 days of the COVID-19 infection. The drugs used by at least 50 users were included. The results were controlled in the multivariable model including age and sex. Drugs were classified according to the Anatomical Therapeutic Chemical (ATC) classification system by WHO Collaborating Centre for Drug Statistics Methodology, Norwegian Institute of Public Health.^13^ The ATC-groups and the number of studied drugs in each category are summarized in Table 1.

**Table 1.** Sex and Geographical Distribution

Variable	Survivors (n)	Dead (n)	All (n)	30 days all-cause mortality (%)
Female	3003	167	3170	5.27
Male	2763	149	2912	5.12
Helsinki and Uusimaa	4140	258	4398	5.87
Other locations	1626	58	1684	3.44
All	5766	316	6082	5.20

### Statistical Analyses

Data on diagnoses registered as the basis of reimbursement was used for controlling select findings in multivariable models as indicated in *Results*. For statistical analyses, chi-square and Fisher exact tests were used for comparisons between drug users and non-users. Each drug with unique ATC-code was analysed separately and only drugs with minimum of 50 users in the population were included in the study. Potential associations between the drug use and mortality were analysed in binary logistic regression models, with death as a dependent variable and drug use (purchase before or within 30 days from positive COVID-19 test) as an independent variable. Age at time of positive COVID-19 test and sex were used as controlling variables in all models. SPSS version 27.0 (IBM Corp. Released 2020. IBM SPSS Statistics for Windows, Version 27.0. Armonk, NY: IBM Corp) and R 4.0.3 (R Core Team (2020). R: A language and environment for statistical computing. R Foundation for Statistical Computing, Vienna, Austria. URL https://www.R-project.org/.) softwares were used for analyses.

## Results

The study cohort included 6082 PCR-confirmed COVID-19 cases. Males represented 47.9% of the study cohort. The mean age of the study cohort was 47.9 (SD 18.8) years. At time of COVID-19 diagnosis, 83.0 % were 18-64 years, and 17.0 % were above 65 years. After selecting those cases that met the previously defined criteria for drug exposure, 4582 patients remained for multivariable models. The mean age at time of positive COVID-19 test was 47.9 years (in survivors 46.0 years and in those who died 82.4 years, p<0.001). Sex and geographical distribution and their effect on mortality (p=0.790 and <0.001, respectively) in the study cohort is presented in Table 1. Mortality was higher in Helsinki and Uusimaa compared to other regions. The number of drugs associated with lower and higher 30-days all-cause mortality after positive COVID-19 test in each ATC group is shown in Table 2. The 73 prescribed drugs included in the analysis and their ATC-codes are presented in Table 3.

**Table 2.** ATC-Groups and the Number of the Studied Drugs in Each Category

ATC-group	Name	Number of the studied drugs	Number of the drugs associated to lower 30 days all-cause mortality	Number of the drugs associated to higher 30 days all-cause mortality
A	Alimentary tract and metabolism	13	0	6
B	Blood and blood forming organs	6	0	1
C	Cardiovascular system	12	3	3
D	Dermatologicals	1	0	0
G	Genitourinary system and sex hormones	2	0	0
H	Systemic hormonal preparations excluding sex hormones and insulins	2	0	0
J	Anti-infectives for systemic use	4	0	0
L	Antineoplastic and immunomodulating agents	0	0	0
M	Musculoskeletal system	2	0	0
N	Nervous system	20	0	7
P	Antiparasitic products, insectisides and repellents	1	0	0
R	Respiratory	8	0	0
S	Sensory	2	0	0
V	Various	0	0	0

**Table 3.** Drugs Included in the Analyses

Name	ATC-code	N	Odds ratio	95 % CI	P Value
Amlodipine	C08CA01	345	1.13	0.77-1.66	0.537
Apixaban	B01AF02	95	1.54	0.94-2.54	0.089
Artificial tears etc.	S01XA20	153	1.17	0.69-1.99	0.551
Acetylsalicylic acid	B01AC06	88	1.72	1.03-2.88	**0.039**
Atorvastatin	C10AA05	292	1.30	0.88-1.92	0.193
Betamethasone (for topical use)	D07AC01	59	0.56	0.15-2.05	0.381
Bisoprolol	C07AB07	449	1.42	1.03-1.95	**0.031**
Buprenorphine	N02AE01	73	1.92	1.11-3.32	**0.020**
Calcium and vitamin D	A12AX	272	1.05	0.72-1.52	0.805
**Candesartan**	**C09CA06**	**205**	**0.34**	**0.16-0.72**	**0.005**
Chloramphenicol (ophtalmological)	S01AA01	122	0.38	0.12-1.19	0.096
Cefalexin	J01DB01	210	0.48	0.27-1.79	0.074
Cetirizine	R06AE07	172	0.48	0.18-1.26	0.136
Citalopram	N06AB04	61	0.50	0.18-1.42	0.194
Clopidogrel	B01AC04	59	0.91	0.47-1.79	0.794
Codeine-paracetamol	N02AJ06	174	0.65	0.23-1.82	0.412
Diclofenac	M01AB05	99	2.72	0.87-8.48	0.085
Donepezil	N06DA02	53	1.57	0.86-2.87	0.140
Doxycycline	J01AA02	193	1.14	0.55-2.37	0.725
Empagliflozin	A10BK03	52	3.32	1.38-8.00	**0.007**
Enalapril	C09AA02	83	2.08	1.13-3.82	**0.019**
Enoxaparin	B01AB05	170	0.70	0.37-1.31	0.262
Erdosteine	R05CB15	82	1.21	0.54-2.72	0.645
Escitalopram	N06AB10	124	1.49	0.70-3.17	0.305
Esomeprazole	A02BC05	88	1.72	1.03-2.88	**0.039**
Estradiol	G03CA03	195	0.63	0.28-1.43	0.269
Fluticasone	R03BA05	73	1.57	0.43-5.69	0.494
Fluticasone (nasal)	R01AD08	60	0.69	0.08-5.95	0.734
Fluticasone (nasal combination preparations)	R01AD58	173	1.15	0.39-3.44	0.801
Furosemide	C03CA01	222	1.65	1.15-2.37	**0.007**
Ibuprofen	M01AE01	708	0.83	0.40-1.70	0.602
Insulin aspart	A10AB05	51	0.94	0.32-2.76	0.913
Insulin glargine	A10AE04	107	1.86	1.00-3.46	0.051
Ispaghula	A06AC01	54	1.26	0.47-3.37	0.647
Lactulose	A06AD11	53	3.62	1.88-6.97	**<0.001**
Levothyroxine natrium	H03AA01	361	1.11	0.73-1.70	0.627
Losartan	C09CA01	194	1.06	0.64-1.74	0.825
Losartan and diuretics	C09DA01	63	0.94	0.33-2.72	0.910
Macrogol	A06AD15	65	3.14	1.69-5.84	**<0.001**
Melatonin	N05CH01	213	1.25	0.81-1.95	0.314

Name	ATC-code	N	Odds ratio	95 % CI	P Value
Memantine	N06DX01	150	1.47	0.99-2.19	0.057
Metformin	A10BA02	250	1.33	0.84-2.10	0.224
Metoclopramide	A03FA01	69	5.34	2.36-12.10	**<0.001**
**Metoprolol**	**C07AB02**	**71**	**0.37**	**0.16-0.88**	**0.024**
Metronidazole	P01AB01	58	2.70	0.78-9.38	0.117
Mirtazapine	N06AX11	224	1.50	0.99-2.26	0.054
Montelukast	R03DC03	101	1.05	0.39-2.85	0.920
Olanzapine	N05AH03	62	2.16	1.08-4.30	**0.029**
Oxazepam	N05BA04	140	2.59	1.48-4.52	**<0.001**
Oxycodone	N02AA05	148	3.09	2.06-4.63	**<0.001**
Pantoprazole	A02BC02	493	1.34	0.95-1.88	0.095
Paracetamol	N02BE01	1082	1.33	0.98-1.79	0.065
Pivmecillinam	J01CA08	89	1.10	0.52-2.34	0.796
Potassium chloride	A12BA01	80	1.48	0.83-2.65	0.180
Prednisolone	H02AB06	140	1.54	0.85-2.81	0.154
Pregabalin	N03AX16	79	1.74	0.90-3.36	0.101
Quetiapine	N05AH04	134	2.33	1.39-3.91	**0.001**
Ramipril	C09AA05	166	1.13	0.70-1.82	0.625
Risperidone	N05AX08	93	1.46	0.88-2.42	0.142
Rivaroxaban	B01AF01	59	0.85	0.39-1.85	0.680
**Rosuvastatin**	**C10AA07**	**143**	**0.44**	**0.21-0.94**	**0.034**
Salbutamol	R03AC02	531	1.16	0.71-1.90	0.561
Salmeterole-Fluticasone	R03AK06	73	1.31	0.54-3.16	0.554
Simvastatin	C10AA01	218	0.94	0.61-1.43	0.766
Sitagliptin	A10BH01	50	3.66	1.56-8.59	**0.003**
Tamsulosin	G04CA02	74	1.47	0.78-2.77	0.230
Temazepam	N05CD07	62	1.50	0.68-3.33	0.316
Tramadol	N02AX02	71	1.61	0.55-4.70	0.386
Trimethoprim	J01EA01	58	1.76	0.66-4.68	0.257
Valproic acid	N03AG01	64	1.45	0.67-3.14	0.346
Venlafaxine	N06AX16	68	3.28	1.37-7.89	**0.008**
Warfarin	B01AA03	72	0.73	0.39-1.37	0.323
Zopiclone	N05CF01	100	2.22	1.15-4.27	**0.018**

Three drugs associated with lower 30 days all-cause mortality among adult COVID-19 infected patients (Table 3): candesartan (aOR 0.34, 95% CI 0.16-0.72, p=0.0047), metoprolol (aOR 0.37, 95% CI 0.16-0.88, p=0.024) and rosuvastatin (aOR 0.44, 95% CI 0.21-0.94, p=0.034).

## Discussion

The COVID-19 patients who used either candesartan, metoprolol or rosuvastatin, had significantly lower 30-days mortality rate compared with COVID-19 patients not using these drugs.

Candesartan has been in the focus of the scientific research from the very beginning of the COVID-19 pandemic for its’ potential protective effect against severe COVID-19, although there are no recommendations for using candesartan against COVID-19 outside its’ normal cardiovascular indications.^14,15^ In line with our results, in a Chinese study, candesartan displayed a better effect in treating patients with COVID-19 and cardiovascular diseases than other angiotensin receptor blockers.^15^ A recent study from Spain reported that candesartan has a protective effect against COVID-19 mortality when used for its normal cardiovascular indications.^12^

Metoprolol is a betablocker that has been proposed to be useful in critically ill COVID-19 patients.^16^ In that pilot trial, intravenous metoprolol administration to patients with COVID-19-associated adult respiratory distress syndrome seemed to reduce exacerbated lung inflammation and improve oxygenation, possibly due to its’ capacity to calm down the ongoing cytokine storm.^17-19^ As far as we are aware, this is the first study, which shows an association between metoprolol and COVID-19 mortality.

Rosuvastatin is used for secondary prevention of cardiovascular disease in patients diagnosed with hypercholesterolemia.^20^ Statins are lipid-lowering drugs with pleiotropic antithrombotic, anti-inflammatory and immunomodulatory effects.^21^ It has also been hypothesized that statins might have impacts to the course of COVID-19 infection due to their ability to downregulate the receptors for angiotensin converting enzyme 2, potentially leading to decreased virus entry to cells. A study from Hubei province, China, showed reduced mortality amongst the statin-users hospitalized for COVID-19 infection.^22^ Previous data reported by Davoudi et al suggests that simvastatin use is associated to less severe COVID-19 illness. They found no correlation between the COVID-19 disease severity and rosuvastatin use, however, the number of rosuvastatin users included in the study was low.^23^ However, Sandhu et al and Israel et al have reported protective effect of pre-existing rosuvastatin medication against COVID-19 hospitalization.^24,25^ In our study, the use of rosuvastatin but not the other statins, was associated with lower 30-days all-cause mortality among COVID-19 patients.

We consider that our study cohort is rather representative because it included all PCR-confirmed COVID-19 cases in adults during the study period, before the COVID-19 vaccines were available. Still, at that time the testing capacity was insufficient, and it is likely that in our study the COVID-19 negative group includes some truly COVID-19 positive cases. At the study time frame, the population in Finland was subjected to relatively strict infection control measures, making extensive and unrecognized spreading of the virus in the population unlikely. Based on serological surveys, 0.5-1% (but not more than 1.5%) of the population became infected in the Helsinki-Uusimaa region (a hot spot for the virus spreading during the first wave) by the beginning of the July 2020.^26^ Higher mortality in Helsinki and Uusimaa compared to other regions (Table 1) may be linked to relatively higher healthcare burden as this region was a hot spot for the virus spreading.

In the present study we used prescription drug purchases as an estimate of prescription drug use. It is generally known that not all the patients receiving a prescription for a certain medication are truly using it. Therefore, we selected using the prescription drug purchases, instead of data on prescribed drugs for analyses. We assume that the drug purchases are generally a good estimate for the true prescription drug use. As the data on prescription drug redemptions are automatically transferred to the national register, it will provide a comprehensive data set for the analysis. However, there are some exceptions, like pain-killers paracetamol and ibuprofen, that are commonly purchased without prescription. In case of these drugs, the register data is likely to underestimate their use.

A clear limitation in our study was that the registers used did not include comprehensive data on the other diagnoses except for COVID-19. We were unable to analyse the effects of chronic diseases, such as for example type 2 diabetes, hypertension, cardiovascular disease, COPD, cancer or kidney failure, known to associate to higher COVID-19 mortality, to the results. However, the reported protective associations of cardiovascular drugs candesartan, rosuvastatin and metoprolol upon their use according to their normal cardiovascular indications are unlikely to be biased by this in a clinically significant way. Instead, the present study design reports the protective effects of these three cardiovascular drugs in a real-world population which makes the results better generalizable to other populations. Still, the lack of comprehensive diagnosis data would significantly complicate evaluation of any potential associations of the drugs to increased COVID-19 mortality, as the underlying diseases may bias the results to unknown extent. Notably, two diabetes drugs, sitagliptin and empagliflozin, both previously suggested to have protective effects against COVID-19 mortality, were associated to higher mortality in the present study. Previous data on the effects of two diabetes drugs on COVID-19 mortality is controversial.^27-29^ Recently, the role of gut-brain axis and effects of microbiome to COVID-19 disease manifestations has gained increasing interest.^30^ Several drugs have been shown to interfere with the gut microbiome and thereby to have complex effects to the gut-brain axis and immune system.^31,32^ The connection between the alimentary tract and nervous system drugs and COVID-19 related mortality may suggest role of gut-brain axis dependent underlying mechanisms and warrants further studies.

## Conclusions

In conclusion, the COVID-19 patients who used either candesartan, metoprolol or rosuvastatin had significantly lower 30-days all-cause mortality rate than non-user controls. Further studies are needed to explore the causes of death in the users and non-users of these drugs to better understand the mechanisms of the protective effects. Also, more research is needed to show whether the protective effects of candesartan, metoprolol and rosuvastatin against 30-days mortality among unvaccinated COVID-19 patients will be repeated in vaccinated COVID-19 patients. Favoring these drugs associated with lower mortality over other drug options could help to further reduce COVID-19 associated mortality of patients with cardiovascular diseases in a population level.

## Data Availability

The research permission does not allow sharing the data outside the research group defined in the permission. If one would like to use the data for research purposes, research permission for this should be applied from the Finnish Social and Health Data Permit Authority Findata.*
